# Trends in Early Outpatient Drug Therapy in Pediatric Inflammatory Bowel Disease in Finland: A Nationwide Register-Based Study in 1999–2009

**DOI:** 10.5402/2012/462642

**Published:** 2012-08-16

**Authors:** Lauri J. Virta, Kaija-Leena Kolho

**Affiliations:** ^1^Research Department, The Social Insurance Institution (Kela), 20720 Turku, Finland; ^2^Children's Hospital, Helsinki University Central Hospital, University of Helsinki, P.O. Box 281, 00029 Helsinki, Finland

## Abstract

*Objective*. There are limited data on the changes of treatment strategies of disease-modifying drugs used to treat pediatric inflammatory bowel disease (IBD). *Methods*. We utilized data from two national registers: the Drug Reimbursement Register for drug costs (for identifying children with IBD) and the Drug Purchase Register (for exposure to drugs), both of which are maintained by the Social Insurance Institution of Finland. The frequencies and trends of drug therapy strategies during the first year of pediatric IBD were evaluated between 1999 and 2009. *Results*. A total of 481 children diagnosed with IBD were identified. During the first six months, 68% of the patients purchased systemic corticosteroids; these combined with 5-aminosalicylic acid in almost all cases. The use of corticosteroids was stable from the early years compared with the end of the study period. In Crohn's disease, there was a trend towards more active use of azathioprine: the therapy was introduced earlier and proportion of pediatric patients purchasing azathioprine increased by up to 51% (*P* < 0.05). *Conclusions*. In pediatric IBD, the majority of patients purchased corticosteroid within the first six months, reflecting moderate-to-severe disease. During recent years in pediatric Crohn's disease, the therapeutic strategies of oral medication have changed towards more active immunosuppression with azathioprine.

## 1. Introduction

An increase in the incidence of pediatric inflammatory bowel disease (IBD), namely, Crohn's disease and ulcerative colitis (UC), has recently been observed in many Western countries, including the Nordic countries [1–4]. It has been estimated that there are about 1–1.5 million patients with IBD in the United States. The prevalence of pediatric IBD being approximately 71 per 100, 000 the treatment of this disease burdens significantly the health care [5, 6]. Medical specialists agree that the pediatric form of the disease has a more aggressive disease course than IBD in adults [7]. This is reflected in the high use of corticosteroids and in the high frequency of surgeries [8]. There is, however, no evidence that the disease phenotype has changed along with increasing incidence of the disease. In many countries, most pediatric patients have Crohn's disease [3], but in Finland UC is about twice as common as Crohn's disease [4]. In children, Crohn's disease in most cases affects the colon and it is uncommon for the disease to be limited to the upper gastrointestinal tract or to the small intestine only [9]. 

The recent guidelines for treating pediatric Crohn's disease [10] or UC [11, 12] are mainly based on data extrapolated from adult studies [13–15]. Of the immunomodulators, azathioprine is used most often, whereas the use of methotrexate has been limited [16]. The use of the TNF-*α* antagonist agent infliximab has been approved for moderate-to-severe Crohn's disease in children older than six years of age in 2006 and restricted to hospital use. Infliximab was approved for clinical use in pediatric UC in 2011 but not yet in younger children. The other TNF-*α* antagonist agent, adalimumab, has been used for study purposes [17, 18], but, likewise, it has not yet been approved for clinical use in pediatric IBD. There has been, however, lively discussion on whether or not IBD therapy should be shifted towards more active immunosuppression by the time of diagnosis to ensure a complication-free disease course [19, 20] but so far there is no clear consensus on the criteria for introducing immunomodulators at an early phase of pediatric IBD. It is possible, however, that the therapeutic strategies of pediatric IBD have begun using immunomodulation more widely. Likewise, it is unclear whether the availability of TNF-*α* antagonists has changed the pattern of therapeutic strategies. This is important to assess for the future when the impact of immunosuppression on the long-term prognosis of the pediatric patients will be an important issue. 

Here, we used the opportunity to study drug purchases in pediatric IBD patients during the first year after diagnosis using nationwide data from comprehensive registers. We studied trends in treatment strategies between 1999 and 2009 and in the periods of 1999-2005, 2006–2007 and 2008-2009.

## 2. Subjects and Methods

### 2.1. Data Sources

In Finland, all IBD patients, irrespective of the place of residence and the socioeconomic status of the family, are entitled to special refunds governed by the Social Insurance Institution of Finland (Kela) to cover part of the medical costs. Kela processes drug reimbursements according to a written certificate describing the diagnostic criteria for IBD (ICD-10 code K50 or K51) signed by a specialist in pediatrics and/or gastroenterology. The certificates are checked by a medical examiner (physician) before Kela grants the reimbursement. Rejections are exceptional, particularly for children, and reimbursements are included in the routine care of the patients. Using the Drug Reimbursement Register, we identified 481 Finnish children aged 0–15 years born between 1 January 1994 and 31 December, 2008 who had been diagnosed with IBD by 30 September, 2009. Kela's administrative process takes only a couple of weeks. Therefore, the date of the special refund decision was defined as the index date of diagnosis. 

We identified drug purchases, a proxy indicator for outpatient drug use during the first year after receiving a diagnosis (followup until September 2010), using the nationwide Drug Purchase Register, which lists drugs according to the World Health Organization's Anatomical Therapeutic Chemical (ATC) Classification System. The register covers outpatient drug purchases in pharmacies but not the use of such drugs in hospitals. Thus, we could not trace the use of the TNF-*α*-antagonist agent infliximab and, because adalimumab is not yet licensed for pediatric patients, it is rarely used in children. The oral medications included corticosteroids, sulfasalazine, 5-aminosalicylic acid (5-ASA), preparation mesalazine, azathioprine, methotrexate, locally active corticosteroids, local 5-ASA preparations and the antimicrobials: metronidazole and ciprofloxacin. According to the Finnish regulations on reimbursement, the maximum number of tablets supplied covers a three-month period. Thus, most patients purchase the drugs used for maintenance medication more than once a year. We studied trends in therapeutic strategies between 1999 and 2009 for the whole study period and for three specific periods of time: 1999–2005, 2006–2007 and 2008–2009.

### 2.2. Statistical Analyses

We used logistic and linear regression analyses when studying the purchases made by the groups of patients with Crohn's disease and UC or those made during different time periods. As the mean ages of the patients increased towards the end of the study period (with the groups for the years 2006–2007 and 2008–2009 being slightly older than the group for the years 1999–2005), we analyzed the associations between the time periods after adjusting them to account for the differences in age. The statistical significance was set at the 5% level (two-sided). The statistical analyses were performed using the SAS system for Windows (version 9.2 SAS Institute Inc., Cary, NC, USA). 

### 2.3. Ethics

The ethical committee of Kela's Research Department approved the study protocol. In accordance with Finnish regulations, no informed consent is required for registry-based studies in which no contact has been made with the subjects of the study.

## 3. Results

Between 1999 and 2009, we identified a total of 481 children aged 0–15 years with newly diagnosed IBD (incident cases). Of these, 293 had UC and 188 had Crohn's disease. At the onset of disease, the mean age of the patients with UC was 7.9 years (SD 3.9), significantly lower than for those with Crohn's disease (9.3 ± 3.9 years, *P* < 0.001). The majority of the patients were boys: 64% of patients with Crohn's disease and 52% of patients with UC (*P* = 0.010). 

During the first 12 months of the disease, 5-ASA and corticosteroids (both for the systemic use) were the two drugs that patients purchased the most throughout the entire study period (1999-2009). 5-ASA was used with comparable frequencies for Crohn's disease (93%; Table 1) and for UC (94%; Table 2). The proportion of 5-ASA users increased linearly (*P* = 0.040) up to 96% for Crohn's disease in 2008–2009. Accordingly, the proportion of 5-ASA users with UC increased towards the end of the study period from 89% between 1999 and 2005 to 97% between 2008 and 2009 (*P* = 0.028; Table 2). Nearly 75% of IBD patients purchased oral corticosteroids without any significant difference between the subtypes of diagnoses, (Tables 1 and 2). As a rule, corticosteroids were introduced by the time of the diagnosis and only 4.5% of the first-year users made their first purchases more than six months after the diagnosis. The frequency of corticosteroid use stayed constant during the seven-year period (between 1999 and 2005) and the last two years of the study period (2008 and 2009) (Tables 1 and 2). 

Few of the patients with Crohn's disease (20%) purchased locally active therapeutic agents (5-ASA or corticosteroids), whereas nearly half of the UC patients (48%) purchased such agents (*P* < 0.001, age adjusted; Tables 1 and 2). During the first 12 months, the use of two antimicrobials metronidazole and/or ciprofloxacin targeted at microbiota in the gastrointestinal tract was more frequent among patients with Crohn's disease (47%) than among UC patients (31%, *P* = 0.009, age adjusted). Both groups used the drug metronidazole most frequently, using it nearly twice as frequently as ciprofloxacin.

During the first 12 months of the disease, the most widely used immunomodulator was azathioprine. Its use was more frequent among patients with Crohn's disease (43%) than among UC patients (30%; *P* = 0.037, age adjusted; Tables 1 and 2). With Crohn's disease, there was a significant linear trend (*P* = 0.030) to more frequent use of azathioprine over the three study periods (1999-2005, 2006-2007 and 2008-2009), with it reaching 51% for patients diagnosed in 2008–2009 (Table 1). Such an increase in use was not observed in patients with UC; among UC patients, the proportion of users was 35% during the last study period (2008-2009), which was significantly less than for those with Crohn's disease (*P* = 0.033, age adjusted; Tables 1 and 2). 

There was also a significant difference in the time intervals from the diagnosis to the introduction of azathioprine: during the first 12 months after diagnosis, azathioprine was introduced approximately one month earlier to patients with Crohn's disease (113 days) than to those with UC (mean 153 days, *P* = 0.035, age adjusted). Moreover, for patients with Crohn's disease these time intervals decreased towards the end of the study period: the first purchases occurred at a mean of 160 days after diagnosis between the years 1999 and 2005, 123 days between the years 2006 and 2007, and 98 days between the years 2008 and 2009 (*P* for linear trend 0.057). For patients with UC, the corresponding figures were 148, 161, and 146 days. The use of methotrexate was rare. 

During the first year after the diagnosis, the purchases of biologic agent for outpatient use were infrequent; in 2008–2009 only three children with Crohn's disease purchased adalimumab. During the first year of the disease, there were no purchases of mercaptopurine, cyclosporine, tacrolimus or mycophenolate. All of the children purchased some drugs during the first six months, and only 4.2% of patients (Crohn's disease *n* = 9; UC *n* = 11) made no purchases between 7 and 12 months. 

The early (up to the first six months) drug therapy strategies, whether as a single therapy or a combination of therapies, are shown in Table 3. During the first six months, there was a significant linear trend (*P* = 0.027) over the three study periods towards more active use of azathioprine among patients with Crohn's disease, but not among patients with UC. The strategies for IBD pharmacotherapy were associated with the subtype of diagnosis, with the age of the patient at the time of the diagnosis, and with the later years of the patient during the study period (Table 4). Azathioprine was more often prescribed to older patients than 5-ASA. Gender had no significant effect on the pharmacotherapy strategies.

## 4. Discussion

We presented here nationwide, register-based data on the purchases of therapeutic agents by patients with pediatric IBD in Finland during the first year of diagnosis throughout the first decade of the twenty-first century. There are no equally comprehensive reports on the practice of medical management and its changes in pediatric IBD. Clinical guidelines for the treatment of pediatric IBD are sparse [10, 11, 21]; based on limited evidence [12], and the types of therapies vary widely. Among patients recently diagnosed with Crohn's disease, there are statistically significant variation in initial management between different specialist clinics, as is evident in examples from the United States [22]. Our results show that the use of azathioprine in pediatric Crohn's disease has become more commonplace. Most patients were introduced to corticoids within three months of the diagnosis and we observed no significant changes in the proportion of patients purchasing corticoids. Although the nationwide use of the TNF-*α* antagonist infliximab could not be evaluated here, it seems that its availability has not markedly influenced the strategies of the early medical management of pediatric IBD.

5-ASA was the most widely used outpatient therapeutic agent for patients with UC and Crohn's disease; more than 90% of the patients used it. This is significantly more frequent than in the recent report by Hyams et al. (60%) on early drug use, which focuses on the use of thiopurines for patients with pediatric UC [23]. The incidence of UC in Finland is far more common that of Crohn's disease and, as with most pediatric patients, Crohn's disease most often affects the colon or ileocolon [4, 8]. Therefore, it is not surprising that patients with Crohn's disease also frequently use 5-ASA, although the evidence shows that the overall benefits are weak [10, 12]. Patients with UC used systemic 5-ASA therapy more towards the end of the study period: 97% of them did so by the end of the study. This may reflect the high number of patients with pancolitis [8].

In line with recent reports (based on chart review), doctors often prescribe corticoids after first diagnosing IBD. Here, three out of four patients purchased corticoids within the first three months. This is in line with a recent report from Denmark [24] and previously published reports from the IBD registry [25, 26]. During the eleven-year period, there was no significant change in the proportion of patients receiving corticoids, and the figures were comparable between Crohn's disease and UC. Thus, it is unlikely that there has been any significant change in the disease phenotype and severity at one year after diagnosis, although the incidence of pediatric IBD has dramatically increased [3, 4].

There is no guideline yet for introducing azathioprine to patients in the early stages of Crohn's disease, although it is agreed that extensive disease or severe growth failure is an indication of active immunosuppression during the initial course of the disease [10]. With UC, there are no guidelines for azathioprine use in pediatric patients. An increase in the use of immunomodulators for pediatric IBD from 1990 to 2000 was reported in a questionnaire survey of pediatric gastroenterologists, although the response rate was disappointingly only 39% [27] There was a significant increase in the use of azathioprine during the study period and, by the end of the study period, up to 50% of patients with Crohn's disease purchased azathioprine within the first year. In comparison, this therapy was introduced significantly earlier in the years 2008-2009, when the mean time from the diagnosis to the introduction of azathioprine was approximately three months. There were no such changes for patients with UC, and by the end of the study approximately every third patient was using azathioprine. Of the other immunomodulators, the numbers of purchases were low.

Gender had no effect on the treatment strategies, but the use of azathioprine was less frequent in younger patients than in older patients. This may reflect a fear of the side effects of azathioprine or the prolonged use of corticoids. The use of corticoids did not, however, vary according to age. Importantly, although the use of azathioprine significantly increased during this decade, it did not prevent patients from using other therapeutic agents during the first year of diagnosis.

### 4.1. Strengths and Limitations

The main strength of this study was the use of comprehensive nationwide, register-based data on drugs dispensed at pharmacies [28]. Whereas, by focusing on purchases as a monitor of patient drug use, we could not assess patient non-compliance, but in a study like ours there is no recall bias and the data suggest a better correspondence to real use than data based on chart reviews or prescriptions [29]. The disease severity or the clinical disease activity could not be assessed. The focus, however, was on therapeutic strategies and possible changes during the study period rather than on individual therapeutic responses. Furthermore, 98% of the IBD cases in the Finnish Drug Reimbursement Register met modern diagnostic criteria, indicating excellent specificity [4]. All diagnoses were based on written certificates, including the diagnostic criteria, and checked by Kela. Thus, the possibility of misdiagnoses is low. The results of corticosteroid use were somewhat hampered by the fact that 5 mg tablets of prednisolone are not reimbursed in Finland during the years 2006-2007 and, thus, such purchases were not registered. This is unlikely to cause a major bias because the proportion of corticoid use remained fairly constant. 

As a limitation, we could not assess the nationwide use of infliximab, which is licensed for use in pediatric Crohn's disease in children older than six years of age. There were, however, no patients without medication during the first six months after diagnosis, and, during the latter part of the year, patients with no medication were an exception. Thus, the use of infliximab occurs in combination with the more conventional medication and that, evidently, patients solely on infliximab therapy were rare during the first year of diagnosis. This is supported by data gathered for this time period in our hospital, which show that between 2005 and 2010, only 11% of pediatric patients were on infliximab during their first year of diagnosis, and single therapy with infliximab was the exception. Elsewhere in Finland, the total use of infliximab has been less frequent (K.-L. Kolho, unpublished data).

In conclusion, there is limited data on strategies of drug therapy in pediatric IBD. This study demonstrates a high use of corticoids in pediatric Crohn's disease and UC at the time of diagnosis. During the last decade, the therapeutic strategy of Crohn's disease has shifted towards more active use and azathioprine is introduced earlier. So far, the use of biologicals is not reflected in therapeutic strategies for patients during the first year of pediatric Crohn's disease. Whether the increased use of immunosuppressants is efficient in reducing the high surgery rates of pediatric IBD patients needs to be assessed in the future. 

## Figures and Tables

**Table 1:** Pediatric patients with Crohn's disease with purchases of disease modifying drugs for inflammatory bowel disease during the first 12 months by three different periods between the years 1999 and 2009.

Medication		1999–2009		1999–2005		2006-2007		2008-2009		*P* value^a^
	ATC-code	*n* = 188	(%)	*n* = 38	(%)	*n* = 53	(%)	*n* = 97	(%)	for linear trend
5-ASA (acting locally)	A07EC02	25	(13.3)	7	(18.4)	8	(15.1)	10	(10.3)	0.222
5-ASA (for systemic use)	A07EC02	174	(92.6)	32	(84.2)	49	(92.5)	93	(95.9)	0.040
Sulfasalazine	A07EC01	3	(1.6)	1	(2.6)	0		2	(2.1)	0.879
Corticosteroids (acting locally)	A07EA02; A07EA06	19	(10.1)	8	(21.1)	5	(9.4)	6	(6.2)	0.059
Corticosteroids (for systemic use)	H02AB06; -02; -04; -07; -09; A07EA06	137	(72.9)	29	(76.3)	36	(67.9)	72	(74.2)	0.802^b^
Metronidazole	P01AB01	74	(39.4)	16	(42.1)	15	(28.3)	43	(44.3)	0.414
Ciprofloxacin	J01MA02	36	(19.1)	5	(13.2)	7	(13.2)	24	(24.7)	0.131
Azathioprine	L04AX01	80	(42.6)	12	(31.6)	19	(35.8)	49	(50.5)	0.030
Methotrexate	L04AX03; L01BA01	6	(3.2)	2	(5.3)	0		4	(4.1)	0.947

^a^Adjusted for age. ^b^Corticosteroids were analyzed periods 1999–2005 versus 2008-2009 (nonlinear trend over all three periods) as between the years 2006 and 2007 prednisolon 5 mg tablets were not reimbursed in Finland and, thus, a portion of data was not available.

**Table 2:** Pediatric patients with ulcerative colitis with purchases of disease modifying drugs for inflammatory bowel disease during the first 12 months by three different periods between the years 1999 and 2009.

Medication		1999–2009		1999–2005		2006-2007		2008-2009		*P* value^a^
	ATC-code	*n* = 293	(%)	*n* = 109	(%)	*n* = 86	(%)	*n* = 98	(%)	for linear trend
5-ASA (acting locally)	A07EC02	84	(28.7)	21	(19.3)	30	(34.9)	33	(33.7)	0.261
5-ASA (for systemic use)	A07EC02	276	(94.2)	97	(89.0)	84	(97.7)	95	(96.9)	0.700
Sulfasalazine	A07EC01	25	(8.5)	12	(11.0)	6	(7.0)	7	(7.1)	0.314
Corticosteroids (acting locally)	A07EA02; A07EA06	96	(32.8)	38	(34.9)	29	(33.7)	29	(29.6)	0.406
Corticosteroids (for systemic use)	H02AB06; -02; -04; -07; -09; A07EA06	211	(72.0)	75	(68.8)	58	(67.4)	78	(79.6)	0.078^b^
Metronidazole	P01AB01	78	(26.6)	15	(13.8)	29	(33.7)	34	(34.7)	0.473
Ciprofloxacin	J01MA02	32	(10.9)	2	(1.8)	17	(19.8)	13	(13.3)	0.494
Azathioprine	L04AX01	89	(30.4)	20	(18.3)	35	(40.7)	34	(34.7)	0.245
Methotrexate	L04AX03; L01BA01	7	(2.4)	2	(1.8)	2	(2.3)	3	(3.1)	0.333

^a^Adjusted for age. ^b^Corticosteroids were analyzed periods 1999–2005 versus 2008-2009 (nonlinear trend over all three periods) as between the years 2006 and 2007 prednisolon 5 mg tablets were not reimbursed in Finland and, thus, a portion of data was not available.

**Table 3:** Drug therapy strategies during the first 6 months among pediatric patients with inflammatory bowel disease (*n* = 481) by three different periods between the years 1999 and 2009.

Medication	1999–2005		2006-2007		2008-2009		*P* value^a^
	*n*	(%)	*n*	(%)	*n*	(%)	for linear trend
Crohn's disease							
Local therapy only (5-ASA and/or corticosteroid)	2	(5.3)	0		0		
Single therapy with 5-ASA	10	(26.3)	15	(28.3)	20	(20.6)	
Single therapy with corticosteroid	3	(7.9)	1	(1.9)	0		
Combination therapy with 5-ASA and corticosteroid	16	(42.1)	21	(39.6)	34	(35.1)	
Combination therapies with azathioprine^b^	7	(18.4)	16	(30.2)	43	(44.3)	0.027

In total	38	(100.0)	53	(100.0)	97	(100.0)	

Ulcerative colitis							
Local therapy only (5-ASA and/or corticosteroid)	2	(1.8)	0		2	(2.0)	
Single therapy with 5-ASA	39	(35.8)	29	(33.7)	18	(18.4)	
Combination therapy with 5-ASA and corticosteroid	52	(47.7)	36	(41.9)	58	(59.2)	
Combination therapies with azathioprine^b^	16	(14.7)	21	(24.4)	20	(20.4)	0.776

In total	109	(100.0)	86	(100.0)	98	(100.0)	

^a^Adjusted for age; trend was not analyzed for corticosteroid and 5-ASA because prednisolon 5 mg tablets were not reimbursed in Finland between the years 2006 and 2007. ^b^Including systemic 5-ASA or/and corticosteroid.

**Table 4:** Background data related to the systemic drug therapy strategies during the first 6 months of pediatric inflammatory bowel disease (*n* = 471 patients).

	Single		Combinations				
Variable	5-ASA		5-ASA + corticosteroid		Azathioprine^a^		*P* value
	*n* = 131		*n* = 217		*n* = 123		
Gender (male), *n* (%)	79	(60.3)	116	(53.5)	71	(57.7)	0.435
Age at IBD diagnosis, mean (SD)	7.5	(3.8)	8.6	(3.9)	9.4	(3.8)	<0.001
Subtype (diagnosis of Crohn's disease), *n* (%)	45	(34.4)	71	(32.7)	66	(53.6)	<0.001
Time period (years 2008-2009), *n* (%)	38	(29.0)	92	(42.4)	63	(51.2)	0.002

^a^With other medications (e.g., 5-ASA or/and corticosteroid).

## References

[1] Hildebrand H, Finkel Y, Grahnquist L, Lindholm J, Ekbom A, Askling J (2003). Changing pattern of paediatric inflammatory bowel disease in Northern Stockholm 1990–2001. *Gut*.

[2] Perminow G, Brackmann S, Lyckander LG (2009). A characterization in childhood inflammatory bowel disease, a new population-based inception cohort from South-Eastern Norway, 2005–07, showing increased incidence in Crohn’s disease. *Scandinavian Journal of Gastroenterology*.

[3] Benchimol EI, Fortinsky KJ, Gozdyra P, Van den Heuvel M, Van Limbergen J, Griffiths AM (2011). Epidemiology of pediatric inflammatory bowel disease: a systematic review of international trends. *Inflammatory Bowel Diseases*.

[4] Lehtinen P, Ashorn M, Iltanen S (2011). Incidence trends of pediatric inflammatory bowel disease in Finland, 1987–2003, a nationwide study. *Inflammatory Bowel Diseases*.

[5] Kappelman MD, Porter CQ, Galanko JA (2011). Utilization of healthcare resources by U.S. children and adults with inflammatory bowel disease. *Inflammatory Bowel Diseases*.

[6] Kappelman MD, Rifas-Shiman SL, Kleinman K (2007). The prevalence and geographic distribution of Crohn’s disease and ulcerative colitis in the United States. *Clinical Gastroenterology and Hepatology*.

[7] Griffiths AM, Buller HB, Walker WA, Durie PR, Hamilton JR, Walker-Smith JA, Waktinks JB (2000). Inflammatory bowel disease. *Pediatric Gastrointestinal Disease*.

[8] Turunen P, Ashorn M, Auvinen A, Iltanen S, Huhtala H, Kolho K-L (2009). Long-term health outcomes in pediatric inflammatory bowel disease: a population-based study. *Inflammatory Bowel Diseases*.

[9] Heyman MB, Kirschner BS, Gold BD (2005). Children with early-onset inflammatory bowel disease (IBD): analysis of a pediatric IBD consortium registry. *Journal of Pediatrics*.

[10] Van Assche G, Dignass A, Reinisch W (2010). The second European evidence-based consensus on the diagnosis and management of Crohn’s disease: special situations. *Journal of Crohn’s and Colitis*.

[11] Tomomasa T, Kobayashi A, Ushijima K (2004). Guidelines for treatment of ulcerative colitis in children. *Pediatrics International*.

[12] Wilson DC, Thomas AG, Croft NM (2010). Systematic review of the evidence base for the medical treatment of paediatric inflammatory bowel disease. *Journal of Pediatric Gastroenterology and Nutrition*.

[13] Travis SPL, Stange EF, Lémann M (2008). European evidence-based Consensus on the management of ulcerative colitis: current management. *Journal of Crohn’s and Colitis*.

[14] Kornbluth A, Sachar DB, Practice Parameters Committee of the American College of Gastroenterology (2010). Ulcerative colitis practice guidelines in adults: American College of Gastroenterology, Practice Parameters Committee. *American Journal of Gastroenterology*.

[15] Mowat C, Cole A, Windsor A (2011). Guidelines for the management of inflammatory bowel disease in adults. *Gut*.

[16] Ruemmele FM (2009). Immunomodulation with methotrexate: underused and undervalued?. *Digestive Diseases*.

[17] Martín-de-Carpi J, Pociello N, Varea V (2010). Long-term efficacy of adalimumab in paediatric Crohn’s disease patients naïve to other anti-TNF therapies. *Journal of Crohn’s and Colitis*.

[18] de Bie CI, Escher JC, de Ridder L (2012). Antitumor necrosis factor treatment for pediatric inflammatory bowel disease. *Inflammatory Bowel Diseases*.

[19] Devlin SM, Panaccione R (2009). Evolving inflammatory bowel disease treatment paradigms:top-down versus step-up. *Gastroenterology Clinics of North America*.

[20] Burger D, Travis S (2011). Conventional medical management of inflammatory bowel disease. *Gastroenterology*.

[21] Turner D, Travis SPL, Griffiths AM (2011). Consensus for managing acute severe ulcerative colitis in children: a systematic review and joint statement from ECCO, ESPGHAN, and the porto IBD working group of ESPGHAN. *American Journal of Gastroenterology*.

[22] Kappelman MD, Bousvaros A, Hyams J (2007). Intercenter variation in initial management of children with Crohn’s disease. *Inflammatory Bowel Diseases*.

[23] Hyams JS, Lerer T, Mack D (2011). Outcome following thiopurine use in children with ulcerative colitis: a prospective multicenter registry study. *American Journal of Gastroenterology*.

[24] Jakobsen C, Munkholm P, Paerregaard A, Wewer V (2011). Steroid dependency and pediatric inflammatory bowel disease in the era of immunomodulators—a population-based study. *Inflammatory Bowel Diseases*.

[25] Markowitz J, Hyams J, Mack D (2006). Corticosteroid therapy in the age of infliximab: acute and 1-year outcomes of newly diagnosed children with Crohn’s disease. *Clinical Gastroenterology and Hepatology*.

[26] Hyams J, Markowitz J, Lerer T (2006). The natural history of corticosteroid therapy for ulcerative colitis in children. *Clinical Gastroenterology and Hepatology*.

[27] Markowitz J, Grancher K, Kohn N, Daum F (2002). Immunomodulatory therapy for pediatric inflammatory bowel disease: changing patterns of use, 1990–2000. *American Journal of Gastroenterology*.

[28] Furu K, Wettermark B, Andersen M, Martikainen JE, Almarsdottir AB, Sørensen HT (2010). The Nordic countries as a cohort for pharmacoepidemiological research. *Basic and Clinical Pharmacology and Toxicology*.

[29] Beardon PHG, McGilchrist MM, McKendrick AD, McDevitt DG, MacDonald TM (1993). Primary non-compliance with prescribed medication in primary care. *British Medical Journal*.

